# Mismatch Negativity in Recent-Onset and Chronic Schizophrenia: A Current Source Density Analysis

**DOI:** 10.1371/journal.pone.0100221

**Published:** 2014-06-20

**Authors:** W. Ross Fulham, Patricia T. Michie, Philip B. Ward, Paul E. Rasser, Juanita Todd, Patrick J. Johnston, Paul M. Thompson, Ulrich Schall

**Affiliations:** 1 Centre for Translational Neuroscience and Mental Health, The University of Newcastle, Newcastle, New South Wales, Australia; 2 Schizophrenia Research Institute, Darlinghurst, New South Wales, Australia; 3 Hunter Medical Research Institute, Newcastle, New South Wales, Australia; 4 School of Psychology, The University of Newcastle, Newcastle, New South Wales, Australia; 5 School of Psychiatry, University of New South Wales, Sydney, New South Wales, Australia; 6 Schizophrenia Research Unit, South Western Sydney Local Health District, Sydney, New South Wales, Australia; 7 Department of Psychology and York Neuroimaging Centre, University of York, Heslington, United Kingdom; 8 Imaging Genetics Center, Institute for Neuroimaging and Informatics, Keck School of Medicine, University of Southern California, Los Angeles, California, United States of America; 9 Departments of Neurology, Psychiatry, Radiology, Engineering, Pediatrics, and Ophthalmology, University of Southern California, Los Angeles, California, United States of America; Centre Hospitalier Universitaire Vaudois and University of Lausanne, Switzerland

## Abstract

Mismatch negativity (MMN) is a component of the event-related potential elicited by deviant auditory stimuli. It is presumed to index pre-attentive monitoring of changes in the auditory environment. MMN amplitude is smaller in groups of individuals with schizophrenia compared to healthy controls. We compared duration-deviant MMN in 16 recent-onset and 19 chronic schizophrenia patients versus age- and sex-matched controls. Reduced frontal MMN was found in both patient groups, involved reduced hemispheric asymmetry, and was correlated with Global Assessment of Functioning (GAF) and negative symptom ratings. A *cortically-constrained* LORETA analysis, incorporating anatomical data from each individual's MRI, was performed to generate a current source density model of the MMN response over time. This model suggested MMN generation within a temporal, parietal and frontal network, which was right hemisphere dominant only in controls. An exploratory analysis revealed reduced CSD in patients in superior and middle temporal cortex, inferior and superior parietal cortex, precuneus, anterior cingulate, and superior and middle frontal cortex. A region of interest (ROI) analysis was performed. For the early phase of the MMN, patients had reduced bilateral temporal and parietal response and no lateralisation in frontal ROIs. For late MMN, patients had reduced bilateral parietal response and no lateralisation in temporal ROIs. In patients, correlations revealed a link between GAF and the MMN response in parietal cortex. In controls, the frontal response onset was 17 ms later than the temporal and parietal response. In patients, onset latency of the MMN response was delayed in secondary, but not primary, auditory cortex. However amplitude reductions were observed in both primary and secondary auditory cortex. These latency delays may indicate relatively intact information processing upstream of the primary auditory cortex, but impaired primary auditory cortex or cortico-cortical or thalamo-cortical communication with higher auditory cortices as a core deficit in schizophrenia.

## Introduction

Deficits in auditory processing as indexed by mismatch negativity (MMN), an electrophysiological response to deviant stimuli, are consistently reported in schizophrenia [1], [2]. However, the underlying neural generators of MMN and how they are functionally related to neuropathology, symptomatology and functioning in schizophrenia remain open to debate [3].

MMN is a component of the event related potential (ERP) to deviant auditory stimuli. It is typically measured in a scalp electroencephalogram (EEG) recorded during presentation of an unattended auditory oddball paradigm in which rare deviant stimuli are randomly delivered within a stream of common standards, with stimulus deviance being defined by changes to the intensity, pitch, or duration of a pure tone [1]. MMN peaks about 150 ms after stimulus deviance and is seen as a negative potential, broadly distributed over frontal sites, with a positive phase reversal over the mastoids when using a nose reference.

The original cognitive model of MMN envisaged it as a preattentive aspect of the orienting response in which incoming auditory stimuli are contrasted to a sensory memory trace of past stimuli, and stimulus deviance above a threshold triggers reallocation of attention [4]. While this model is still current, several alternative models have been derived from it. The auditory system may maintain a *predictive* model of the acoustic environment derived from regularities in the acoustic input [5], [6]. Violations of the model, which occur when the current sensory input does not match model predictions, lead to model updating and generation of MMN. Several variants of this theory use a Bayesian statistical framework to describe the predictive function, and the updating, of this internal model of the sensory world [7].

### Abnormal MMN in schizophrenia

Reduced MMN in schizophrenia was first demonstrated in medicated chronic patients, using duration-deviant tones [8]. Subsequent research demonstrated that the MMN reduction cannot be attributed to neuroleptic medication [9], [10] and remains relatively stable between the acute and non-acute phases of the disorder in chronic patients [11]. MMN reduction displays some degree of selectivity for schizophrenia compared to other diagnoses with overlapping symptomatology, such as bipolar affective disorder [9] and major depression [12], though this has been challenged by recent studies [13], [14] and a major review indicating that MMN may index general cognitive decline within a broad spectrum of clinical disorders [3]. While there have been mixed reports about the correlation between reduced MMN amplitude and symptom ratings [1], there is a well-replicated association with lower scores on the Global Assessment of Functioning (GAF) scale [15]–[19], and with other measures of social and occupational function [20]–[22]. Analyses of brain structure in schizophrenia show correlations with loss of grey matter, especially near primary auditory cortex [22], [23].

Given the demonstrated reliability of smaller MMN amplitude in patients compared to controls [10], [24], and early reports of smaller MMN amplitude in their biological relatives [25], there has been substantial interest in the extent to which this may be considered a trait, or possibly an endophenotype [24], of schizophrenia. Of particular interest is whether a similar pattern occurs during the prodromal phase of schizophrenia. Preliminary studies show smaller duration MMN amplitude occurs in at-risk groups [26]–[28], and predicts conversion to a schizophrenia diagnosis, whilst pitch MMN does not distinguish between converters and non-converters [29]. However, these observations of smaller MMN amplitude in the prodromal phase are challenged by studies showing intact MMN in first episode patients [23], [30] and mixed findings for MMN in patients' biological relatives [31]. These inconsistencies may arise partly because duration MMN deficits are seen early in the illness, whereas pitch MMN tends to be intact at first-episode and reduced only in chronic schizophrenia [27], [32].

In the current study, we do not directly assess MMN in prodromal schizophrenia. However by examining MMN in recent-onset patients (on average ten months after first onset) and in chronic patients, we can gain some insight into the progression of MMN changes and their association with psychopathology and functioning. Further, detailed modelling of the cortical generators of the MMN signal may generate novel insights into the neuropathology of schizophrenia.

### Cortical Generators of MMN

The scalp-recorded MMN has multiple cortical generators. An early response is generated in or near the primary auditory cortex and in the immediately surrounding secondary auditory cortex in the posterior superior temporal gyrus and *planum temporale*. A later, but temporally overlapping, response is generated in either the middle or inferior frontal gyrus, particularly in the right hemisphere [33]–[36]. Given the location and orientation of these sources, it has been argued that in nose-referenced EEG, the MMN recorded at the mastoids primarily indexes the temporal response, while the MMN recorded at frontal sites receives contributions from both temporal and frontal generators. The temporal generator has been associated with auditory feature analysis and deviance detection, and the frontal generator with the involuntary switching of attention towards changes in the auditory environment [37]. This argument is supported by observations of the functional dissociation of the mastoid and frontal MMN in a variety of experimental paradigms, for example manipulation of deviant probability [38], and effects of low dosage ethanol [39].

Identification of the neural generators in this model is derived from converging evidence from a variety of neuroimaging procedures. However, each imaging procedure has certain limitations. The original proposal suggesting temporal and frontal sources was made by Näätänen and Michie [4] and reasserted by Giard et al. [40] who identified two sources in their scalp current density (SCD) maps. However, SCD has low spatial resolution, cannot discriminate nearly adjacent sources, and is relatively insensitive to deep or broadly distributed sources. Early MEG studies, using equivalent current dipole (ECD) modelling, identified a single temporal source in or near Heschl's gyrus, i.e., primary auditory cortex [41]. MEG is optimally sensitive to tangentially oriented sources such as those in the superior temporal plane (STP), but is blind to radial sources. This has been suggested as the reason MEG consistently fails to detect the frontal source [35]. Positron Emission Tomography (PET) and functional Magnetic Resonance Imaging (fMRI) studies have identified superior temporal gyrus and inferior frontal sources [36], but these are embedded in a broader network that includes cingulate, cuneus, parietal, lingual, and hippocampal regions [42], [43]. However, PET and fMRI lack temporal resolution so it is difficult to determine whether the observed clusters of activity are associated with the MMN, or with later ERP components such as the P3a [43]. The temporal source has also been identified using electrocorticograms in patients with epilepsy undergoing presurgical planning [44], [45]. However, due to the clinical nature of these studies they provide only limited data concerning sources in other cortical regions.

Attempts to directly *model* the scalp-recorded MMN using discrete equivalent current dipoles (ECD) invariably locate bilateral sources in the vicinity of the superior temporal gyrus. When additional unconstrained dipoles are incorporated in these models extra-temporal sources have variously been reported in right frontal cortex [36], right inferior/middle frontal cortex [46]–[49], left anterior cingulate [46]–[49], right medial frontal cortex [50], and the right inferior parietal cortex [51]. Across these studies, at least part of the variability in the observed source locations might be attributable to differences in the number of, and the constraints imposed on, sources within the models used. Solutions of discrete ECD models are particularly sensitive to misspecification of the number of dipolar sources, and the solutions obtained become less robust in the presence of noise as the number of model dipoles increases [52].

Given the suggestion from fMRI and PET studies of broadly distributed cortical MMN activation, Current Source Density (CSD) analysis may provide a more valid modelling approach [52]. Using CSD, the EEG is modelled by a very large array of current source dipoles distributed throughout the brain, making few a priori assumptions about the number and locations of cortical sources. LORETA [53] is a widely-used form of CSD analysis that imposes a smoothness constraint on the model solution, and which has been applied to the study of MMN in controls [54] and in schizophrenia patients [55]. LORETA analyses are often performed using a generic head model with a regular grid of ECD sources distributed throughout the brain volume (or limited to a presumed grey-matter region) without orientation constraints [53]. However, further refinements of this approach are possible that allow individual variability in cortical anatomy, especially the orientation of cortical surfaces, to be included within the model. In the present study, each individual's MRI data are used to define a realistic head model within which the entire cortical surface is represented. For this *cortically-constrained* LORETA analysis, the grid of current sources is constrained to lie on, and be perpendicular to, the cortical surface, and all model calculations are performed within the individual's native brain space [56].

Similar cortically-restrained CSD methodology has been applied in two previous MMN studies. Rinne et al. [35] examined pitch MMN in healthy individuals using both EEG and MEG. They observed temporal and frontal sources within individuals' EEG data but only the temporal source was detected using MEG. However, they were unable to create group-averaged CSD maps and were thus less able to detect weaker or more distributed sources. They performed a Region of Interest (ROI) analysis that split frontal from posterior-temporal cortex and demonstrated that the frontal source peaked substantially later than the temporal response. Park et al. [57] proposed an extended methodology that mapped each individual's CSD solution into a common brain space, thus permitting group averaged statistical comparisons using the voxel-based procedures commonly used in fMRI and PET studies [58]. They illustrated this procedure by examining pitch MMN in controls and chronic schizophrenia patients. At the peak of the MMN response, cortical sources were observed in a left hemisphere dominant distributed network, including the STG and large areas of the parietal cortex. Schizophrenia patients exhibited CSD reductions in the left STG and inferior parietal regions. Controversially, these results and those in a companion paper [59] were challenged [60], [61] largely because both the parietal response and the left hemispheric dominance were inconsistent with previous findings. In the current study, we adopted a similar CSD method to that proposed by Park et al., although we employed an alternative approach to control statistical bias in CSD maps.

## Method

### Ethics Statement

Ethics approval for the study was granted by the Human Research Ethics Committees of the University of Newcastle, the University of New South Wales, the Hunter New England Health District, and the South Western Sydney Local Health District. Written informed consent was obtained from all participants.

### Participants

Participant groups consisted of 16 individuals with recent-onset schizophrenia (duration of illness less than 2 years from their first psychotic episode); 19 individuals with chronic schizophrenia (duration of illness greater than 5 years); and two groups of 16 and 19 individually age- and sex-matched controls. Within this study, we use the term *chronic* only to indicate length of time since first treatment onset, rather than to imply any additional diagnostic criteria. Participants with schizophrenia were out-patients, tested in remission whilst on maintenance levels of antipsychotic medication. Exclusion criteria for all participants included neurological conditions (history of major head injury, stroke or epilepsy), significant hearing loss (>20 dB between 500–2,000 Hz), recent history of substance abuse including cannabis, and standard MRI exclusion criteria. Additional exclusion criteria for controls included a current or lifetime diagnosis of a psychotic disorder or family history of schizophrenia.

Recent-onset schizophrenia participants were recruited through two early psychosis services based at a metropolitan and a large regional hospital and their associated hospital wards. Chronic schizophrenia participants were recruited through outpatient sources, including the Schizophrenia Research Institute's (SRI) volunteer register. Control participants were recruited from the SRI register, hospital staff, and university students.

Participant details are summarised in Table 1. Patients had lower educational levels than controls. As has frequently been reported, patients smoked more cigarettes than controls, with the rate of smoking being particularly high in the chronic patient group. The clinical status of the patient groups is summarised in Table 2. The recent-onset and chronic patient groups had a mean duration of illness of one year and 15 years, respectively. Both groups contained a small number of unmedicated individuals, with the remainder taking a variety of antipsychotic and antidepressant medication. Note that only chronic patients were taking any typical antipsychotic medications. Age at onset of first psychotic episode differed between the recent-onset and chronic groups (22 and 24 years respectively). This measure was based on a self-report within the chronic patient group, so it might reflect a reporting-bias rather than necessarily being a sampling-bias between the two groups. The two patient groups did not differ on GAF or negative symptom ratings, but the chronic group had significantly worse positive symptom ratings.

**Table 1:** Description of Participant Groups.

	Recent-Onset		Chronic		Statistical Comparison		
	Control	Patient	Control	Patient	Diagnosis	Illness Duration	Diagnosis x Duration
N	16	16	19	19			
Male:Female	11∶5	11∶5	10∶9	10∶9	*ns*	*ns*	*ns*
Left:Right	1∶15	2∶14	1∶18	4∶15	*ns*	*ns*	*ns*
Newcastle:Sydney	10∶6	5∶11	13∶6	11∶8	*ns*	*ns*	*ns*
Age	24.3(3.2)	22.7(4.2)	38.9(11.0)	40.2(10.0)	*ns*	***	*ns*
Cigarettes	0.6(2.1)	4.6(8.8)	0.6(2.4)	15.0(19.5)	**	*ns*	**
Education	5.1(0.9)	3.2(1.2)	4.9(1.5)	4.0(1.9)	***	*ns*	*ns*

Descriptive information for each group, reported as the mean (and SD), including total numbers, breakdown by sex, handedness and research site (Newcastle, Liverpool); age; Cigarettes smoked per day; and Education Level (1..6). Last three columns summarise results from a diagnosis x illness duration two-way ANOVA or from chi-squared analysis, as appropriate.

*p<.05;

**p<.01;

***p<.001; ns Not Significant.

**Table 2:** Clinical descriptive data for patient groups.

		Recent-Onset	Chronic	*p*
N		16	19	
Medication				
	Unmedicated	5	2	*ns*
	Typical Antipsychotic	0	7^a^	*
	Atypical Antipsychotic	11	13^a^	*ns*
	Antidepressant	6	9	*ns*
Onset Age		22.1(4.2)	24.4(6.5)	*
Duration (yrs.)		.8(.92)^b^	15.8(7.3)^c^	***
GAF				
	Total	55.2(10.0)	53.6(12.0)	*ns*
SAPS				
	Delusions	.52(.53)	1.02(.76)	*ns*
	Hallucinations	.67(.85)	.98(1.0)	*ns*
	Thought Disorder	.27(.50)	.75(.90)	**
	Bizarre Behaviour	.47(.58)	.43(.58)	*ns*
	Total	.48(.35)	.86(.61)	**
SANS				
	Alogia	1.21(.89)	.91(1.0)	*ns*
	Affective Flattening	1.51(.81)	1.71(1.3)	*ns*
	Inappropriate Affect	.80(1.0)	1.21(1.2)	*ns*
	Avolition	2.22(.88)	2.25(1.2)	*ns*
	Anhedonia	2.09(1.1)	2.17(1.5)	*ns*
	Attention	1.31(1.5)	1.35(1.4)	*ns*
	Total	1.63(.75)	1.67(.87)	*ns*

Clinical assessments for patient groups. Medication status and mean (SD) of Age, Duration of Illness, SAPS, SANS and GAF scores. SAPS and SANS scores are scaled to a maximum symptom severity of 5. GAF scores reflect percentage of optimal functioning. Last column contains P values obtained from a t-test or chi-squared analysis, as appropriate, comparing recent-onset to chronic groups.

* *p*<.05;

** *p*<.01;

*** *p*<.001; ns Not Significant.

a Three individuals were taking both typical and atypical antipsychotic medication.

b Range 0.2 to 2 years.

c Range 5 to 29 years.

### Clinical Assessment

A diagnosis of schizophrenia was confirmed using either the Structured Clinical Interview for DSM-IV (SCID) [62] or the Diagnostic Interview for Psychosis (DIP) [63]. Symptom severity was rated using the Scale for the Assessment of Positive Symptoms (SAPS) [64], the Scale for the Assessment of Negative Symptoms (SANS) [65], and the Global Assessment of Functioning (GAF, DSM-IV Axis V) [66].

### Structural MRI

Structural MRIs were acquired using a Siemens Magnetom Vision (Newcastle) or a Siemens Magnetom Symphony (Sydney) 1.5 T whole-body MRI scanner equipped with a Siemens quadrature head coil. A magnetisation prepared rapid acquisition gradient echo (MPRAGE) sequence was employed to acquire a 164-slice T1-weighted anatomical image of the whole head with voxel size of approximately 1 mm^3^. (Siemens Vision: TR = 9.7 ms, TE = 4 ms, flip angle  = 12^°^, 256×256 matrix, FoV = 250 mm; Siemens Symphony: TR = 2000 ms, TE = 3.9 ms, flip angle  = 15^°^, 256×256 matrix, FoV = 256 mm).

### Stimuli

Participants watched a video with muted audio while binaural auditory stimuli were presented using calibrated headphones. Stimuli consisted of 92% standard tones (50 ms, 90 dB SPL, 1000 Hz sine wave, 10 ms rise and fall times) and 8% duration-deviant tones (100 ms). The stimulus sequence was pseudorandom (deviants were preceded by at least one standard) with a fixed SOA of 500 ms. Two blocks of 1250 tones were presented with a short intervening break.

### Electroencephalograph (EEG) recording

EEG data were recorded from 60 scalp sites using an electrode cap (Quick Cap, Neuroscan) and from both mastoids referenced to the tip of the nose. VEOG was recorded from electrodes above and below the left eye. HEOG was recorded from electrodes at the outer canthi of each eye. The EEG was digitised at 500 Hz with a 0.15–30 Hz bandpass and 50 Hz notch-filter using a SynAmps I or SynAmps II EEG system (Neuroscan). Electrode locations were digitised using a Fastrak 3D digitiser (Polhemus).

### ERP Data Analysis

Initial ERP data analysis was performed using Scan v4.3 software (Neuroscan). Continuous EEG records were inspected visually to exclude gross artifact. Bad EEG channels (max. 2 per participant) were replaced by linear interpolation of adjacent channels. Blink artifact was reduced using a linear regression procedure [67]. EEG epochs (400 ms prestimulus to 600 ms poststimulus) containing artifact exceeding ±100 µV were rejected. Standard tones immediately following a deviant stimulus were excluded from further analysis. ERPs to standard and deviant stimuli were obtained by averaging the corresponding EEG epochs.

The MMN was extracted by subtraction of the standard from the deviant ERP, followed by baseline correction over the 200 ms preceding the onset of the difference between the two stimuli. MMN was analysed using the mean amplitude across two 50 ms time intervals (Early MMN: 110–160 ms; Late MMN: 160–210 ms). To permit examination of laterality effects, MMN was assessed at the F3-F4 electrode pair and at the mastoids. In a preliminary analysis, we confirmed that effects observed at Fz were comparable to those at F3–F4.

Each ERP measure was subjected to separate analysis. Patient and control participants were organised into pairs matched on age, sex and, when possible, research institution. Three-way ANOVAs were performed on mean ERP amplitude from electrode pairs with Diagnosis (Schizophrenia, Control) and Hemisphere (Left, Right) as repeated measures factors; and Illness Duration (Recent-Onset, Chronic) as a between-subject factor. Significant interaction effects were examined using simple effects.

Pearson correlations were computed between MMN data, demographic variables, and symptom ratings, using mean amplitudes across F3 and F4 and across M1 and M2 as measures of frontal and mastoid MMN, respectively. Due to the relatively small sample sizes, the two patient groups were combined to form a single group prior to analysis.

### Realistic Head Model

Using Curry v4.6 (Compumedics), for each individual, the EEG electrode grid was coregistered to the structural MRI using three anatomical landmarks (nasion, left and right preauricular points). Realistic head models were extracted as wire-frame surfaces of the scalp, outer- and inner-skull surfaces. The scalp, skull and brain compartments were assigned default values for electrical conductivity (.33, 0042, 33 S/m respectively). Cortical extraction was performed semi-automatically using region growing algorithms. The extracted cortical surface lay midway between the external cortical surface and the grey-white matter boundary so that it bisected an estimate of total cortical grey-matter volume.

### Cortically-Constrained LORETA Current Source Density Analysis

Approximately 17000 equivalent current dipoles were distributed uniformly on the extracted cortical surface. The mean distance between dipoles along the cortical surface was 3.6 mm with each dipole simulating the activity of a cortical patch with a mean area of 10.5 mm^2^. Dipole orientation was constrained to be perpendicular to this surface. Forward calculations of the electric field due to each dipole were performed using the Boundary Element Method. The ERP difference waves were common average-referenced and, for the model fit, were inversely weighted by a noise estimate obtained from the 200 ms interval preceding stimulus deviance. The inverse solution was constrained using a cortical surface LORETA procedure [56] which produces the smoothest possible distribution of current sources across adjacent nodes on the 2D cortical surface consistent with the observed EEG data. For the goodness-of-fit criteria, the ratio between data and model terms was adjusted until the model adequately predicted observed MMN in the grand average at sites located at the periphery of the electrode montage, and was then fixed for all subjects to avoid statistical bias between groups. *Cortically-constrained* LORETA analyses were performed independently for each subject and for each time point in the -150 to 450 ms interval.

The *cortically-constrained* LORETA solution consists of a vector field of current dipoles defined by both amplitude and dipolar orientation at each grid point on the cortical surface. To facilitate averaging across subjects and statistical analysis, only the absolute magnitude of the vector field solution, the current source density (CSD), is analysed, discarding orientation information. CSD at any location is always a positive quantity and will be proportional to both the true ERP signal and to the noise in the ERP signal. This raises two issues. First, under the null hypothesis that there is no true ERP signal, CSD values approach but do not equal zero, making null hypothesis testing difficult. Secondly, since CSD magnitude reflects both ERP signal and ERP noise, larger CSD values are obtained from noisy data. This potentially confounds group comparisons of control versus patients given the possibility that patient data may be noisier than control data. To correct for noise bias, we adopted the following procedure. The original EEG data was reprocessed. After artifact rejection, EEG epochs were randomly assigned to two split-half groups, each containing 50% of the deviant and 50% of the standard trials. One split-half was inverted by multiplying by –1. The full data set was then processed as if it contained normal EEG data. This generates an ERP that contains no MMN response, but which does contain background noise statistically comparable to that present in the original MMN. CSD analysis was then performed on this noise signal as per the MMN analysis. This entire process was repeated twenty times and averaged to produce a stable estimate of the CSD bias for each subject at each dipole and each time point. This bias field was then subtracted from the original CSD to produce a bias-corrected CSD estimate that was used in all further analyses.

### Group Common Brain Space

SPM5 [58] was used to project each individual's structural MRI into a common brain space (MNI305). The sparse grid of CSD results were interpolated to every voxel in this space within 5 mm of the source surface and smoothed using a 10 mm Gaussian kernel. The CSD data at any specific voxel was not normally distributed across subjects, having a large positive skew, especially in regions where the average CSD was maximal. Statistical comparisons between groups were obtained by computing the median CSD at each voxel for both groups, and performing a bootstrap analysis (10,000 replications), to directly estimate the likelihood that the two group medians differed under the null hypothesis. Volume maps of the probability distribution were thresholded at *p* = .001 uncorrected and cluster size above 1 cm^3^.

For patients only, Pearson correlations were performed between CSD and clinical symptoms. For this analysis, the two patient groups were pooled. To minimise the number of comparisons performed, only symptom measures that were significantly correlated with the scalp-recorded MMN were examined. Volume maps were thresholded at *p* = .001 uncorrected and cluster size above 1 cm^3^.

We performed several region of interest (ROI) analyses. ROIs were defined for Heschl's gyrus and the *planum temporale* by manual tracing on individual MRIs. Additionally, three ROIs were defined for middle temporal gyrus, frontal cortex (superior, middle and inferior frontal gyrus), and parietal cortex (cuneus, precuneus, supramarginal gyrus and angular gyrus). These three ROIs were based on the anatomical labelling applied to the Colin27 MRI data set [68] transformed into the anatomical space for each individual. The average CSD activity at cortical surface nodes within each homologous ROI pair was computed as a function of time, and subjected to ANOVA in an identical fashion to the scalp MMN analyses.

### CSD Onset Latency

Onset latencies for activity within each ROI were defined using a segmented regression procedure, specifically the U2df model described by Mordkoff and Gianaros [69], as the point of intersection of two regression lines modelling the prestimulus and rising phases of the MMN response. Segmented regression techniques are frequently used to measure onset latencies of ERP components, especially the lateralised readiness potential, and in simulation studies demonstrate greater sensitivity and less statistical bias than other traditional methods [69], [70]. In applying this approach, we found that signal to noise ratios were too low to permit reliable estimation of onset latency for some individuals. In this situation, it has been demonstrated that greater statistical power can be achieved using jackknife permutation analysis to estimate the reliability of differences between group-averaged waveforms [71]. While generally following this procedure, we elected to alternatively use bootstrap permutations to estimate means and standard errors for onset latencies, and for differences in onset latencies, of group averaged waveforms. T-tests were then computed using the bootstrap estimates to determine the statistical significance of differences in onset latency between participant groups and cortical ROIs.

## Results

### Mismatch Negativity

**Figure 1:**
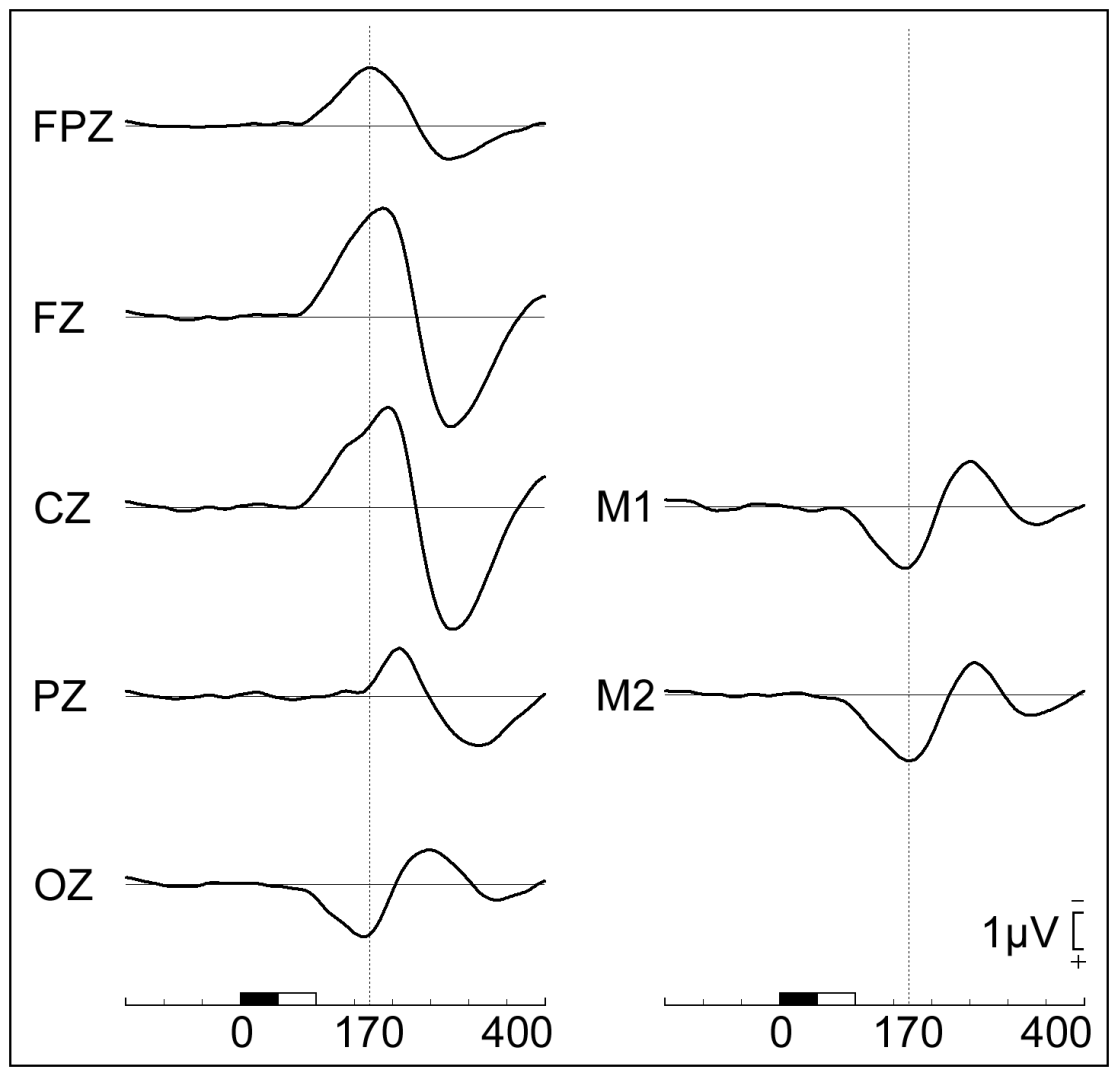
Grand average MMN waveforms. Grand average deviant minus standard difference waves recorded from midline and mastoid sites. The early MMN is maximal at Fz and phase reverses at mastoid sites. Prior to 160

The deviant minus standard difference ERP consisted of an early MMN component which had a midline frontal negativity peaking at approximately 190 ms at Fz, with a phase reversal over bilateral mastoid sites peaking at 170 ms (see Figure 1). This was followed by a temporally- and spatially overlapping late negative component, clearly visible as a separate component at Pz, onsetting at 160 ms and peaking at 210 ms that we label as the late parietal response. This was followed by a large P3a peaking at 275 ms and maximal over FCz.

**Figure 2:**
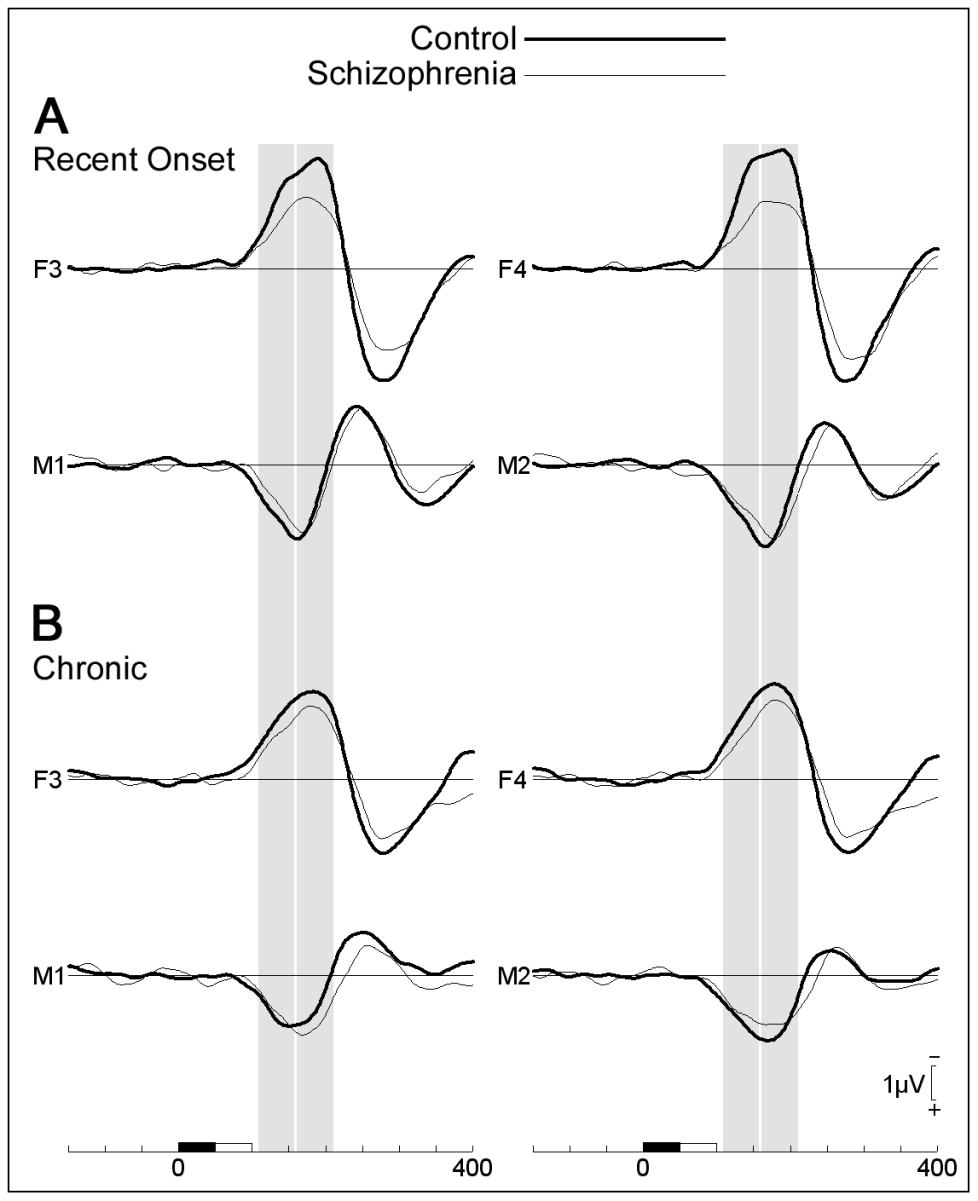
Comparison of MMN at frontal and mastoid sites in controls and patients with schizophrenia. (A) Recent-onset groups; (B) Chronic groups. Shaded vertical bars represent intervals analysed and labelled early and late MMN.

Figure 2 contrasts the MMN waveforms for control and patient groups at the electrode sites analysed. The expected reduction of frontal MMN in schizophrenia is clearly visible, especially for the recent-onset group. However, the magnitude of this effect is smaller than has been reported in some previous studies [1]. In this figure, the two analysis time-intervals that we have labelled as corresponding to the early- and late-MMN are illustrated by vertical grey bars.

Means and standard deviations of the MMN averaged across each time interval are provided in Table S1. Notably, the standard deviations of all measures were similar for control and patient groups. Additionally, although mastoid MMN was smaller than frontal MMN, the standard deviation of these measures was proportional to their amplitude suggesting that frontal and mastoid MMN in this data set have similar signal-to-noise ratios.

### Early MMN (110–160 ms)

At F3-F4, there was a main effect of diagnosis, F(1,33)  = 9.29, p = .005, modulated by an interaction with hemisphere, F(1,33)  = 5.55, p = .025. Patients had smaller MMN than controls over both hemispheres, both *p*s<.02; MMN was larger over the right compared to left hemisphere in controls, F(1,33)  = 9.13, p = .005, but was not lateralised in patients, F(1,33)  = .33, ns. In separate contrasts between each group of patients and their matched controls, recent-onset patients had smaller MMN than controls, F(1,15)  = 5.91, p = .028, but the same trend in chronic patients was not significant, F(1,18)  = 3.24, p = .089. There were no significant effects at the mastoids, all *p*s>.1.

### Late MMN (160–210 ms)

At F3-F4, MMN amplitude was smaller in patients than controls, F(1,33)  = 6.17, p = .018. Interactions with hemisphere and illness duration were not significant. In separate contrasts between each group of patients and their matched controls, recent-onset patients had smaller MMN than controls, F(1,15)  = 4.85, p = .044, but the same trend in chronic patients was not significant, F(1,18)  = 1.49, p = .24.

At the mastoids, there was a main effect of hemisphere, F(1,33)  = 15.1, p<.001; an interaction between diagnosis and hemisphere, F(1,33)  = 4.29, p = .046; and a three-way interaction between diagnosis, hemisphere and illness duration, F(1,33)  = 4.04, p = .052. MMN was larger over the right compared to left mastoid in the recent-onset controls, recent-onset patients and chronic controls groups, F(1,15)  = 10.45, p = .006, F(1,15)  = 5.20, p = .038, F(1,18)  = 8.87, p = .008, respectively, but not in the chronic patients whose response was not lateralized, F(1,18)  = .03, ns.

### Correlations between MMN, Demographic and Clinical measures

There were no correlations, in either patient or control groups, between early or late MMN amplitude and any of the demographic variables including age, years of education, cigarette usage, sex, handedness, or institution at which the research was performed (all *r*s<.30, all *p*s>.05 uncorrected). Additionally, there were no correlations in the patient group with age when diagnosed or duration of illness (all *r*s<.24, all *p*s>.05 uncorrected).

Correlations between MMN amplitude and clinical symptoms are presented in Table 3. Both GAF and negative symptoms were correlated with frontal, but not mastoid, MMN. Smaller MMN was associated with poorer GAF and increased negative symptoms for the total SANS score, and the alogia, affective flattening, avolition and attention sub-scales. There were no significant correlations with positive symptoms.

**Table 3:** Correlations between MMN and Clinical Symptoms.

		Frontal		Mastoid	
		Early	Late	Early	Late
SAPS					
	Delusions	−.22	−.04	−.03	.06
	Hallucinations	−.16	.03	−.21	−.22
	Thought Disorder	.17	.13	−.09	−.13
	Bizarre Behaviour	.13	.10	.13	.11
	Total Score	−.08	.05	−.10	−.08
SANS					
	Inappropriate Behaviour	−.01	.02	.02	.17
	Anhedonia	.24	.14	.11	.07
	Alogia	.42 *	.41 *	.18	.04
	Affective Flattening	.34 *	.39 *	.24	.00
	Avolition	.42 *	.39 *	.16	.22
	Attention	.38 *	.36 *	−.02	.23
	Total Score	.46 **	.44 **	.20	.14
GAF					
	Total Score	−.33 *	−.37 *	−.02	.00

Correlations between GAF, clinical symptoms (SAPS and SANS) and MMN (at frontal and mastoid sites). Negative symptoms correlate with frontal MMN. There were no correlations with positive symptoms, nor with mastoid MMN.

*p<.05 (uncorrected);

**p<.01 (uncorrected).

### Current Source Density Analysis

**Figure 3:**
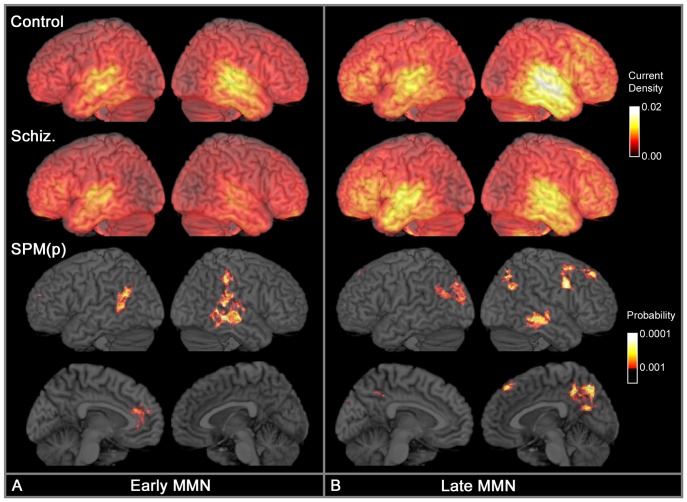
Comparison of CSD in controls and schizophrenia. Comparison of CSD in controls and schizophrenia during (A) Early MMN (110–160 ms), and (B) Late MMN (160–210 ms). Upper two rows display CSD magnitude with maximal activity in lighter colours. CSD has units of micro-Amperes per millimetre squared and has a maximum value of 0.021 µA/mm^2^ in the data illustrated. The medial surfaces are not illustrated as they showed no patterns of focal CSD activity. The lower two rows display clusters in which controls have greater CSD than patients. This is a statistical parameter map (SPM) of the probability that the two groups differ, thresholded at *p*<.001 (uncorrected); cluster size >1 cm^3^. There were no clusters in which patients had larger CSD than controls.

Figure 3 illustrates the bias-corrected CSD associated with the early and late MMN. In controls, CSD was statistically greater than zero (*p*<.001) throughout virtually the entire cortex at both time intervals (not illustrated). Early MMN was associated with focal activity in the posterior dorsal temporal lobe in both hemispheres and a weaker diffuse band of activity extending through parietal cortex. Peak activation was located in the superior temporal sulcus in both hemispheres, with strong activation also in the *planum temporale*. Late MMN was associated with right hemisphere dominant temporal lobe activity; right hemisphere dominant superior, middle and inferior frontal gyrus activity; and weak bilateral parietal activity.

Figure 3 also contrasts control and patient CSD. Visual inspection suggests controls and patients activate similar cortical regions, but the marked right hemispheric dominance seen in controls is absent in patients. Controls had larger CSD than patients in all statistically significant voxels (p<.001 uncorrected). During early MMN, controls had larger CSD than patients in bilateral middle temporal regions. In the right hemisphere, this middle temporal cluster peaked in the superior temporal sulcus and extended posteriorly to include the planum temporale (but not Heschl's gyrus) and the supramarginal gyrus. In the left hemisphere, the middle temporal cluster was more posterior and extended to include the angular gyrus. There was also a small cluster in the left anterior cingulate. During late MMN, in the right hemisphere there was a cluster in the middle temporal gyrus; a large cluster extending through the angular gyrus, superior parietal, superior occipital and precuneus; a cluster in posterior middle frontal and precentral gyrus; and a small cluster in superior frontal cortex. In the left hemisphere, there was a cluster in the posterior middle temporal gyrus extending through the angular gyrus and middle occipital gyrus; and a separate cluster in the middle occipital gyrus. Note that at both latencies examined, differences between controls and patients within temporal cortex were variously identified in regions inferior to, or posterior to, Heschl's gyrus, but none of these clusters included Heschl's gyrus.

### Region of Interest Analysis

Similar patterns of activation were observed in Heschl's gyrus, the *planum temporale*, and middle temporal ROIs. A preliminary omnibus ANOVA including these three ROIs as a repeated factor was performed and revealed no interactions between diagnosis and ROI. Consequently, for the CSD amplitude analysis, these three ROIs were averaged to form a single temporal ROI. Separate ANOVAs were then performed on the temporal, frontal and parietal ROIs.

### Early MMN

CSD was smaller in patients than controls in the temporal ROI, F(1,30)  = 4.43, p = .044; and in the parietal ROI, F(1,30)  = 5.77, p = .023. In the frontal ROI, there was an interaction between diagnosis and hemisphere, F(1,30)  = 5.98, p = 0.021. Simple effects on the frontal ROI revealed no effect of diagnosis within each hemisphere when examined independently, however, controls, but not patients, had larger CSD in the right compared to left hemisphere, F(1,30)  = 4.13, p = .05.

### Late MMN

There were no effects in the frontal ROI. CSD was smaller in patients than controls in the parietal ROI, F(1,30)  = 4.92, p = .034. In the temporal ROI, there was an effect of hemisphere, F(1,30)  = 5.78, p = .023, modulated by an interaction with diagnosis, F(1,30)  = 5.36, p = .028. Simple effects revealed no effect of diagnosis within each hemisphere when examined independently, however, controls, but not patients, had larger temporal CSD in the right compared to left hemisphere, F(1,30)  = 9.18, p = .005.

### ROI Onset Latency

**Figure 4:**
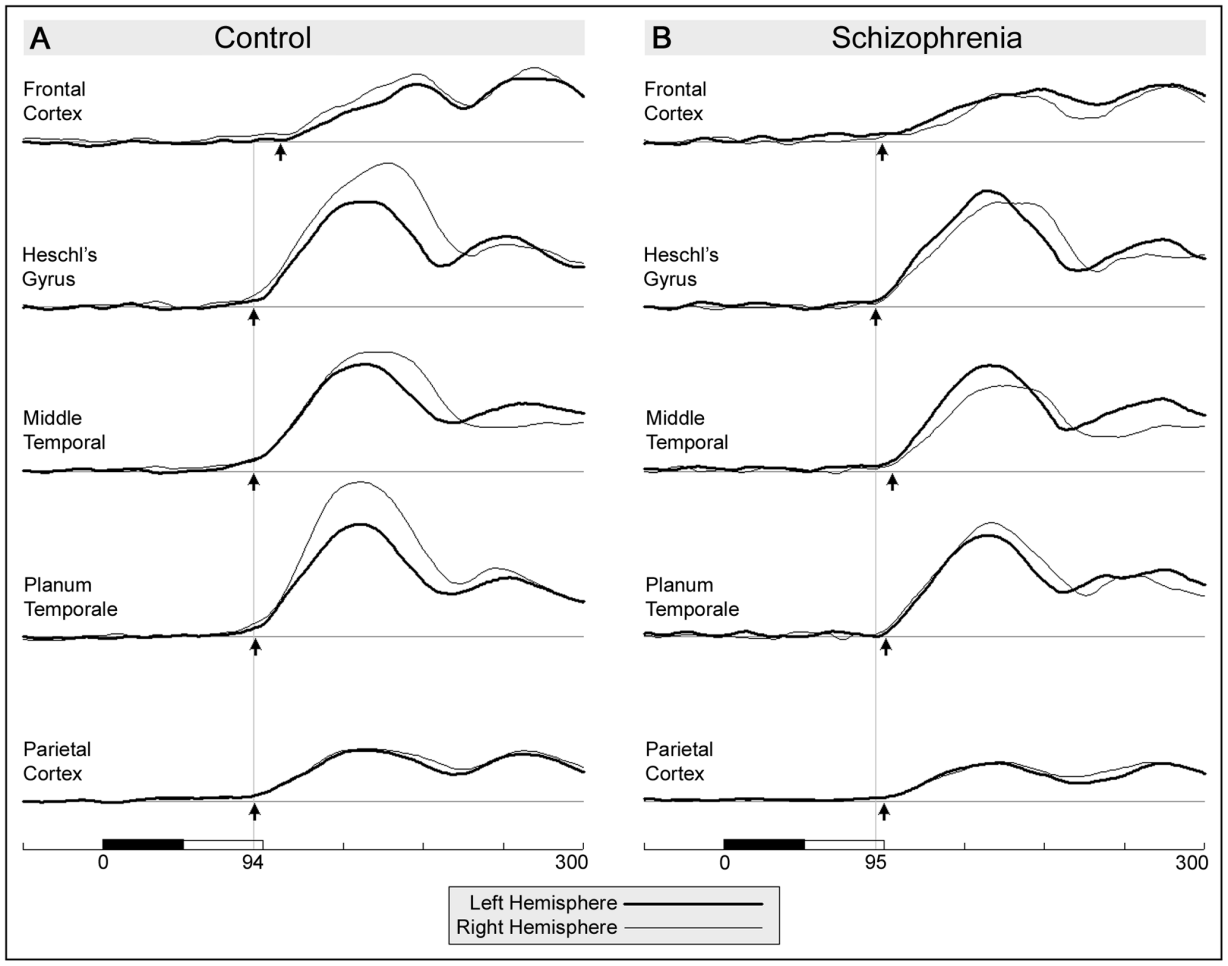
Temporal course of CSD in five ROIs for control and schizophrenia groups. Left hemisphere overlaid on right hemisphere. Small arrows indicate onset latency in each ROI estimated using piecewise linear regression. Vertical lines indicate onset latency in Heschl's gyrus for each group. (A) Average across both Control groups, (B) Average across both Patient Groups.

There were no significant onset latency differences between the hemispheres for any ROI in either group. Consequently, we estimated onset latencies after averaging left and right hemispheres to improve reliability. Figure 4 illustrates the temporal course of CSD in each ROI. Table 4 summarises the onset latency of each ROI for each participant group as well as the group and ROI comparisons. The table additionally reports the standard error of the mean (SEM) for each value derived from the relevant bootstrap analysis. With the exception of the frontal ROI, SEM values did not differ markedly between participant groups or ROIs, so it is unlikely that the different pattern of results in the two participant groups can be attributed to increased variance within the patient group.

**Table 4:** Comparison of CSD Onset Latency across participant groups and cortical regions of interest.

Onset Latency				
	Region of Interest	Control	Schizophrenia	Group Difference
	Heschl's gyrus	94.0 (2.3)	95.3 (3.1)	1.4 (3.9)
	Planum Temporale	95.3 (2.3)	101.2 (2.1)	5.8 (3.2)†
	Middle temporal gyrus	94.5 (3.0)	105.0 (2.6)	10.6 (3.9) **
	Parietal cortex	95.0 (2.6)	100.0 (3.0)	5.0 (4.0)
	Frontal cortex	111.1 (6.7)	98.5 (15.1)^a^	a

Delay relative to Heschl's gyrus				
	Region of Interest	Control	Schizophrenia	Group by ROI Interaction
	Planum Temporale	1.4 (1.3)	5.9 (2.1) **	4.5 (2.4)†
	Middle temporal gyrus	0.5 (2.1)	9.7 (2.5) **	9.2 (3.3) **
	Parietal cortex	1.0 (2.1)	4.6 (4.1)	3.7 (4.8)
	Frontal cortex	17.2 (6.7) *	a	a

The upper portion of this table summarises the onset latency of the CSD within each region of interest for both participant groups. Onset latencies are in milliseconds relative to stimulus onset, which occurs 50 ms before standard and duration deviant stimuli can be differentiated. Values in brackets are the standard error of the mean (SEM) derived from the bootstrap analysis, and not the standard deviation of onset latencies measured from each individual. The right-most column contrasts patient to control data showing the mean difference and the bootstrapped SEM for that comparison. Significance levels were determined using t-tests based upon the bootstrap SEM estimate for that comparison, rather than using a pooled SEM estimate. The lower portion of the table presents the delay in onset latency within each ROI relative to that in Heschl's gyrus. The right-most column contrasts these delays between control and patient groups, and as such measures an interaction between Group and ROI. Excluding the frontal ROI, SEM is relatively consistent across ROIs and participant groups.

† Trend at *p* = .07;

*p<.05;

**p<.01.

a Note variance of the onset latency in frontal cortex for the patient group is excessively large. This measure was excluded from all subsequent analysis and interpretation.

In controls, there were no significant onset latency differences when comparing Heschl's gyrus to the *planum temporale*, middle temporal gyrus, or parietal cortex (all delays <1.4 ms, all *p*s>.28). Onset was delayed by 17.2 ms in frontal cortex relative to Heschl's gyrus, (t(31) = 2.58, p = .015).

In the schizophrenia group, the estimate of onset latency in frontal cortex (98.5 ms, SEM = 15.1) had substantially greater variability than that in all other ROIs for either group and was excluded from all further analysis. Onset delay from Heschl's gyrus to parietal cortex (4.6 ms) was not significant (*p = *.26), but delays to planum temporale (5.9 ms) and middle temporal gyrus (9.7 ms) were significant, (t(31)  = 2.84, p = .008; t(31)  = 3.85, p = .001; respectively).

Onset latency was later in patients than controls in the middle temporal gyrus (D = 10.6 ms, t(61)  = 2.71, p = .009) and as a trend in the planum temporale (D = 5.8 ms, t(61)  = 1.84, p = .07). There were no differences in Heschl's gyrus (D = 1.4 ms) or parietal cortex (D = 5.0 ms).

### Correlations between CSD and Global Assessment of Functioning

**Figure 5:**
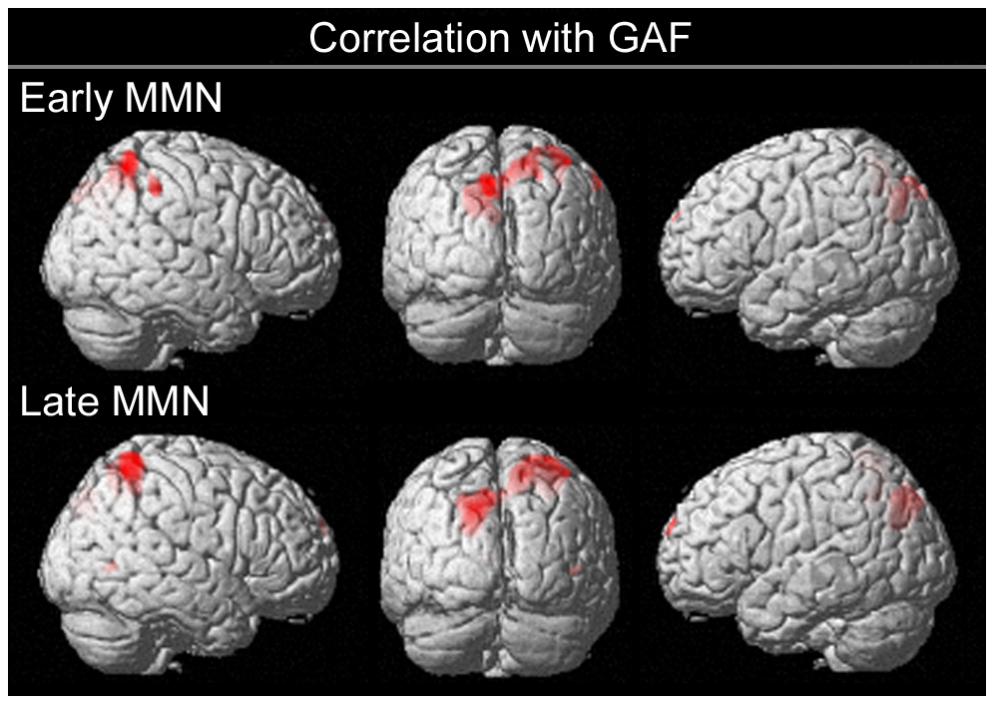
Correlation between GAF and CSD. Pearson correlation between GAF and CSD in schizophrenia patients reveals clusters in bilateral parietal cortex for both early and late MMN time intervals. Figure displays SPM(*t*) thresholded at p<.001 (uncorrected).

For each of the measures of clinical symptoms in patients that showed significant correlations with the scalp-recorded MMN, correlations were performed with the corresponding CSD. GAF was the only measure that showed significant (p<.001, uncorrected) correlations with CSD. Figure 5 illustrates the positive correlation between CSD and GAF scores for the early and late MMN time intervals. For early MMN, clusters occurred in left cuneus/precuneus and right precuneus. For late MMN, a cluster in left precuneus survived voxel-level family-wise error (FWE) correction, *p*_FWE_ = .027; and a cluster in the right superior parietal lobe survived cluster-level FWE correction, *p*_cFWE_ = .026. There were no clusters showing a negative correlation.

We additionally performed a conjunction analysis of the correlation between CSD and GAF with the contrast between control and patient groups, both thresholded at p<.001 uncorrected. For late MMN only, clusters in left precuneus and right superior parietal cortex were present in both contrasts. Thus for these regions, CSD was larger in controls than in patients and was positively correlated with GAF in patients.

## Discussion

### Current Source Density analysis in Controls

Our CSD analysis appears consistent with that reported by Rinne et al. [35] and largely confirms the presence of cortical generators in the temporal and frontal regions. A number of previous EEG and MEG source modelling studies have reported that the principal MMN generator lies in the general vicinity of the superior temporal gyrus [36], [72], [73], and have emphasised the role of auditory cortex located within the superior temporal plane (STP). The STP includes the planum temporale and Heschl's gyrus, and is the superior surface of the temporal cortex largely buried within the Sylvian fissure. Given the location and orientation of the STP, a single equivalent dipole located near primary auditory cortex (i.e. near Heschl's gyrus) has been proposed as a model that explains the phase reversal of the MMN scalp topography between frontal and mastoid electrode sites [1] and has sometimes been used to model the entire MMN response [74]. In our data, we observed activity throughout the superior temporal gyrus, including Heschl's gyrus. However, this activity also spanned the middle temporal gyrus with the maximal CSD being in the superior temporal sulcus (STS) rather than the STP. Using fMRI and PET, clusters of MMN-related activity have been reported in both the STP and STS [36], [75]. This distinction is important, as it implies that a major contributor to the duration MMN signal lies in secondary auditory cortex in addition to any contribution from primary auditory cortex.

During early MMN, activity is seen throughout most of the temporal lobe, extending posteriorly with a weak band of activity in the parietal lobe. As the MMN develops over time, activation in temporal cortex becomes strongly right hemisphere dominant, and spreads broadly throughout the frontal cortex. Although previous reports of a frontal source suggest that it is located in the inferior frontal gyrus [33], [34], [36], [75], our data revealed late activation broadly distributed in frontal regions [42], [43], [76], and centred in the middle frontal gyrus.

Our CSD analysis is consistent with the expected main focus of activity within auditory cortex, whereas other studies using distributed source models have reported maximal activity within left parietal cortex [57], bilateral parietal, visual and sensory-motor cortex [77], and the precentral gyrus [55]. Taken together, these reports suggest that MMN is a part of an extended temporal, frontal and parietal network.

### Onset Latency of Temporal and Frontal sources in Controls

Previous studies have variously reported that the frontal source may peak either before [78]–[81] or after [35], [36], [49], [82] the temporal source. A unique result of the current study is the measurement of the difference in *onset*, rather than peak, latency of the two sources. The onset of the response in Heschl's gyrus occurred 44 ms after the occurrence of physical difference in the deviant and standard stimuli. The onset of the response in the middle frontal gyrus was delayed by an additional 17 ms. This result is consistent with the frontal response being triggered by change-detection processes within auditory cortex. Given this assumption, the observed delay is too large to be accounted for simply by conduction delays along fibre tracts between these regions [83]. Rather it appears more consistent with a delay due to the *sequential* information processing stages within the MMN cortical response.

However, the latency data are not necessarily inconsistent with alternative models of the frontal response. Yago et al. [78] proposed that rather than being triggered by the auditory cortex, the frontal response might be triggered directly by thalamo-cortical pathways arising from subcortical change-detection processes. Under this model, the frontal response might occur either before or after that in auditory cortex. Tse et al. [80] further proposed that the frontal response may contain at least two components. The early frontal component precedes the auditory cortex response and is associated with top-down regulation of the change-detection process, for example contrast enhancement [76] or predictive model generation [5]. The later frontal component is triggered by the change detection process and is associated with processes such as reallocation of attention [4], response inhibition [84], and predictive model updating [5]. Our data provide no evidence of a frontal response that precedes activity within the auditory cortex. However our paradigm does not replicate the conditions under which this has been reported. Tse et al. [80] reported an early frontal response only for small and medium sized deviants, but not for large deviants. Our duration deviant tone was easily discriminable, and thus might not be expected to engage the early frontal component.

### Reduced MMN in Schizophrenia

Our data reveal a smaller duration MMN at frontal electrodes in the recent-onset schizophrenia group compared to healthy controls. These individuals were medicated outpatients in remission, tested within two years of, and on average ten months after, a first episode of psychosis. Our results are consistent with prior reports of reduced duration MMN in recent-onset schizophrenia [26], [32], [85], in acute first-episode schizophrenia [47], and in prodromal and ultra-high risk of schizophrenia groups [26], [29], [72], [86].

The exploratory whole-brain CSD analysis revealed multiple cortical regions in which there was a reduced CSD response in the schizophrenia groups in addition to the expected reductions within auditory cortex. This again supports the view that MMN generators are not confined to auditory cortex. During early MMN, patients had reduced CSD in bilateral posterior temporal cortex, centred over the middle temporal gyrus, including portions of the superior temporal gyrus posterior to Heschl's gyrus, and extending posteriorly to include inferior parietal cortex. There was also a reduction in left anterior cingulum, which has been implicated as an MMN source in EEG studies [46], [55], [87]. During late MMN, in addition to bilateral temporal cortex, reductions within parietal cortex had spread to include the superior parietal lobe, precuneus and regions bordering the occipital lobe. Furthermore there were reductions in right superior and middle frontal lobes consistent with predictions of a right hemisphere-dominant frontal MMN source, although the location of this cluster was more superior than might have been expected.

The region of interest analysis largely reinforced these results, particularly the engagement of the parietal cortex. Of note, the exploratory analysis found reductions throughout most areas of auditory cortex but not in Heschl's gyrus itself. In contrast the ROI analysis suggests that the pattern of reductions observed throughout the planum temporale and the middle temporal gyrus is also present in Heschl's gyrus. Of the two analyses, the ROI analysis has improved anatomical resolution as individual data are not mapped into a common brain space, and it has greater statistical power as it does not need to apply highly conservative statistical thresholds to avoid problems with family-wise error. Thus our data supports the view that CSD reductions occur broadly throughout primary and secondary auditory cortex. This result is consistent with the majority of EEG [49] and MEG [88] source localisation studies, fMRI studies [89], [90], and reports of correlations between reduced grey-matter density within Heschl's gyrus and MMN amplitude in schizophrenia [22], [23].

### Impact of Illness Duration

Several studies have reported that the reduction in duration MMN in chronic patients is *smaller* than the reduction seen in recent-onset patients, the effect being associated with an age-related decline in frontal MMN in healthy controls [91] that is not observed in patients with schizophrenia [32], [47], [92], [93]. In our data, illness duration produced a trend consistent with this pattern, but it was not statistically significant. These reports appear to be contrary to the notion of a frontal MMN deficit that *increases* with the progression of the disorder after first onset as suggested by a meta-analysis of MMN studies published before 2003 [1]. However, that conclusion was influenced by the findings of intact MMN in acute first-episode schizophrenia as well as studies that reported pitch MMN reductions increasing with illness duration.

### Reduced Hemispheric Asymmetry

In controls, the scalp MMN was right-hemisphere dominant at frontal sites during the early MMN and at mastoid sites during the late MMN. This pattern of laterality was also apparent in the CSD analysis in the frontal and temporal ROIs, respectively. In contrast, patient data displayed no hemispheric lateralisation at frontal sites, and only recent-onset patients had normal lateralisation at mastoid sites, though this was not evident in the CSD analysis. Similar findings of reduced hemispheric lateralisation have been reported using MEG [72] and fMRI/MEG [94]. The reduction in hemispheric lateralisation at mastoid sites during the late MMN was the *only* statistically significant effect observed in MMN that was related to illness duration in this study. These changes in lateralisation are more consistent with a degenerative rather than a developmental model of neuropathology in schizophrenia. The reduced hemispheric dominance may be seen as part of a generalised finding of reduced hemispheric specialisation in schizophrenia [95].

### Onset Latency in Heschl's gyrus, Planum Temporale, Middle Temporal and Parietal Cortex

As noted above, a unique feature of this study was the estimation of onset latencies within each of the ROIs examined. The onset latency of a cortical region provides specific information concerning the cortical network that is not easily obtained from the magnitude of the response in that region, nor even from its peak latency. The onset latency of a response primarily reflects the integrity of processes and pathways up-stream from the region examined. In contrast, the amplitude of the response may be influenced by that same upstream process, the integrity of the region itself, or feedback from down-stream regions. Onset latency and amplitude measure are complimentary but independent properties of a cortical network.

In controls, we observed no statistically significant difference in onset latency between Heschl's gyrus, planum temporale, middle temporal and parietal cortex. Given that delays of 6 ms were readily detected within the patient data, any delays that may exist within the control data are likely well below this value. The near simultaneous onsets indicate that these cortical regions are either part of a tightly integrated cortical network with information reaching primary auditory cortex being *rapidly* redistributed via cortico-cortical fibres, or else they form a relatively independent network receiving parallel inputs from a common, probably subcortical, source. The control data are inconsistent with a sequential processing model in which the primary auditory cortex performs *detailed* analysis of the auditory signal before initiating action in the surrounding regions. It seems improbable that each region is independently detecting the specific feature that discriminates standard and deviant stimuli, although it is likely that parallel processing of different stimulus features occur in separate auditory regions. This raises the possibility, at least for duration-deviant stimuli, that change detection, *per se*, is a subcortical process; and that the role of the cortical regions may be to extract detailed information on the deviant stimulus, to enable evaluation of its salience. For more complex stimulus sequences, change detection might only be possible at the cortical level. This proposition is consistent with recent reviews [96], [97] of animal studies and the middle latency auditory response (MLR) in humans that propose a hierarchical change/novelty detection system originating as low as the inferior colliculus (IC) in the midbrain, and medial geniculate nucleus (MGN) in the thalamus. For example, in the cat [98] deviance-related responses are present in the IC and MGN 20 ms before similar responses in auditory cortex. In humans [99], [100], several components of the MLR display genuine deviance-related modulation, possibly in a feature specific fashion [97]. One of these components, Nb, peaks near the onset latency of the MMN, consistent with the suggestion that MMN is part of a cascade of change detection stages. The integrity of deviance modulation within the MLR has yet to be determined in schizophrenia.

In patients, activity in Heschl's gyrus onset at the same latency as seen in controls. However, onset latency in *planum temporale* and middle temporal areas was delayed relative to Heschl's gyrus in the schizophrenia group and was later than that seen in controls. These onset delays are likely to reflect the neuropathology [101] responsible for the bilateral amplitude reductions observed throughout temporal and parietal cortex during the early MMN response. Given the normal onset latency in primary auditory cortex, it would appear that information processing stages upstream of primary cortex are relatively intact. Conversely, the abnormal onset latency in secondary cortex implies a processing deficit upstream of secondary cortex. Note that abnormal feedback from the frontal cortex is probably excluded as the central deficit since the frontal cortex does not begin processing the change-detection signal until well after this point in time. Assuming that information flows sequentially from subcortical regions to primary auditory cortex, and is then distributed to secondary areas (with or without feedback), then a parsimonious interpretation of the data suggests either a processing deficit within primary cortex, or within the cortico-cortical relays between primary and secondary regions. Note that it is possible that the reduced amplitude of the response in primary cortex is a consequence of invalid feedback from secondary regions, rather than a specific problem within primary cortex itself. Consistent with this proposition, impaired feed-forward pathways between lamina *within* primary cortex have been identified in schizophrenia [102], [103]. Also, impairments to feed-forward pathways from primary to secondary auditory cortex have been suggested based on anatomical data [102], models of MMN generation in schizophrenia [104], and models of the effect of ketamine on MMN in healthy subjects [105].

However, auditory pathways from subcortical regions and within the cortex are considerably more complex than that proposed above, giving rise to the possibility of alternative interpretations of the latency data. For example, we might speculate that our data suggest that lemniscal thalamo-cortical pathways leading to primary auditory cortex are intact, while nonlemniscal thalamo-cortical pathways leading to associative auditory cortex are impaired. In a review, Hu [106] notes that lemniscal pathways carry tonotopically-organised auditory-specific information while nonlemniscal pathways form part of an integrative system with roles in polysensory integration and temporal pattern recognition. In particular, nonlemniscal, but not lemniscal, pathways have been associated with stimulus-specific adaptation, a subcortical precursor of mismatch negativity [107]. Note that these proposals are not inconsistent with there being an additional deficit within primary auditory cortex, either intrinsically or as a consequence of lateral interactions with secondary auditory cortex which displayed delayed onsets.

These proposals are speculative. To bolster the argument requires elaboration of the interconnections between subcortical and cortical auditory domains, which are difficult to examine directly in humans. One approach for future research may be the application of dynamic causal modelling (DCM) [6]. DCM provides a Bayesian statistical framework for testing generative neural models of ERPs and has been used to study the pathways between primary, secondary and frontal cortex during MMN [104], [108]. However, to the best of our knowledge, none of the models tested have allowed the possibility of parallel pathways between the thalamus and primary, secondary and frontal cortex; have included both lemniscal and nonlemniscal pathways; nor have been applied to deviance modulation of the MLR.

### Correlation between MMN and Global Assessment of Functioning

Our results replicate previous reports of a robust correlation in schizophrenia between MMN at frontal scalp sites and GAF [15]. Correlations between GAF and CSD identified clusters in bilateral parietal cortex rather than in the auditory or frontal cortex where the major generators of the MMN are purported to be located. The lack of correlation with CSD in auditory cortex is consistent with Pekkonen et al.'s [109] MEG study that found no correlation between duration-deviant MMNm and GAF, as MEG is optimally sensitive only to tangentially oriented sources such as the primary auditory cortex. For future studies examining the relationship between GAF and cognitive function, our results would suggest that GAF might be better predicted by parietal functions (e.g. sensory integration), than by temporal functions (e.g. sensory discrimination) or frontal functions (e.g. executive function).

### Correlation between MMN and Clinical Symptoms

MMN was correlated with negative symptoms including the Alogia, Affective Flattening, Avolition, and Attention SANS sub-scores. As with GAF, these correlations were with the frontal, but not the mastoid MMN, and are consistent with previous reports in chronic schizophrenia [9], [110]–[117]. Correlations in the reverse direction have also been reported [85], [112], [118], but only in studies that included first episode patients. Our results are inconsistent with the conclusion from a meta-analysis performed by Umbricht and Krljes [1] which suggested that MMN does not correlate with clinical symptoms.

We found no correlations between positive symptoms and MMN recorded from either the frontal or mastoid sites. In particular, the data provide no support for prior reports of a relationship between hallucinations and MMN [119], nor for Näätänen and Kähkönen's [120] prediction that positive symptoms would correlate with mastoid MMN in nose-referenced data. However, our participants were all in remission at the time of testing and had relatively low positive symptom ratings, so a floor effect may have limited our ability to detect a correlation with MMN.

### Role of the Parietal Cortex in MMN

Within the CSD analysis, we observed an early parietal response that was both reduced in patients relative to controls and correlated with GAF within the patient group. This would suggest a critical role for parietal cortex in our understanding of MMN reduction in schizophrenia.

However, the observed parietal activation in controls was diffuse rather than focal, was of relatively low amplitude, and onset simultaneously with the substantially larger activity in the immediately adjacent temporal cortex. Given the low spatial resolution of EEG data, the parietal response must be interpreted cautiously. Our CSD analysis employed a cortically-constrained LORETA algorithm, which biases the obtained cortical solution towards the smoothest possible inverse model. Even genuinely focal cortical activity will appear spatially smeared within a LORETA model. Consequently, at least part of the observed parietal CSD may be an artifact of the LORETA procedure attributable to the large response in temporal cortex. Further evidence is required to validate the presence of a separate MMN generator within parietal cortex.

Within our data, we note that (A) differences between patients and controls in parietal cortex were consistently bilateral whereas differences in the temporal and frontal regions were associated with reduced right hemispheric dominance, and (B) parietal cortex was the only region that correlated with GAF. These two results suggest a partial dissociation between the activity in parietal cortex and other regions that would be difficult to explain as a LORETA artifact.

Further support for a parietal source comes from prior MMN studies in controls using intracerebral recordings [44], [121], fMRI [42], [43], [122]–[124], MEG [77], [125]–[127], and EEG [54], [55], [57], [128]. While each of these studies identify a parietal response, it is important to acknowledge that there are also a significant number of similar studies that do not report parietal activity, possibly as a consequence of its relatively low amplitude and diffuse distribution. The cited fMRI papers identify multiple sites within parietal cortex that are differentially activated by deviant stimuli including inferior parietal [42], [43], [123], [124], superior parietal [124] including the precuneus [43], [123], [124], and post-central gyrus [43]. However fMRI lacks the temporal resolution required to verify that this activity occurs during the temporal interval associated with MMN, rather than later time intervals associated with, say, the P3a component. The fMRI study by Salmi et al. [123] reported superior and inferior parietal activation in response to intensity-deviants, and demonstrated that these areas were also activated during top-down (i.e. voluntary) reallocation of attention (See also [129]). This suggests that the parietal activation in these studies is most likely associated with the later phase of the MMN and/or the P3a as part of a fronto-parietal network engaged in the reallocation of attention, rather than as part of the change-detection process per se. This interpretation seems consistent with intracerebral [44], MEG [125] and EEG [54], [128] studies that report *inferior parietal* activity that occurs approximately 50 ms later than the initial change-detection response in auditory cortex, but still within the temporal window associated with the later phase of MMN. The relatively late timing of the parietal response in these studies is inconsistent with our data that shows a parietal response onsetting simultaneously with the change-detection process within auditory cortex and well before the activity in frontal cortex. More consistent with our data, the MEG study by Novitski et al. [126] reports *centro-parietal* activity that occurs simultaneously with that in auditory cortex.

The presumption of a parietal source within our data is substantially due to the observed differences between controls and patients, and to the correlation with GAF scores. We note two prior MMN studies that observed differences between control and schizophrenia patients in parietal cortex. Park et al. [57] observed differences within inferior parietal cortex at locations consistent with our observations in the early MMN time interval. In contrast, Takahashi et al. [55] observed differences within the para-central lobule, which is more consistent with, but anterior to, our observations during the late MMN time interval.

The detection of parietal activity does not appear to be an artifact attributable to a specific source modelling procedure. It has been observed in EEG and MEG studies using scalp current density [128], equivalent current dipoles [125], linear minimum norm estimation [126], Combined ICA-LORETA [54], grey-matter constrained LORETA [57], cortically-constrained LORETA (the current study), eLORETA [55], Multiple Sparse Priors [77], and MEG phase synchronisation [127] analyses.

The parietal cortex subserves multiple functions. Models of auditory perception suggest the presence of a ventral auditory pathway within temporal cortex associated with *identifying* auditory objects, and a dorsal pathway that extends into the parietal cortex associated with *locating* auditory objects in space [130]. Additionally, parietal cortex, and in particular superior parietal cortex, has been implicated in multi-sensory integration [131], merging auditory information with the other sensory domains. Both of these functions are initiated early within the perceptual pathway in a stimulus-driven attention-independent fashion. Parietal cortex is ideally placed to ask a question such as “where did that sound come from and can I see it?” If, as observed in our data, parietal cortex is activated early within the MMN response, then we might *speculate* that this question is being asked not as a consequence of re-orienting towards a stimulus that has been recognised as a deviant, but rather as a mechanism for extracting additional information about the stimulus in order to determine whether or not it *is* a deviant.

In a review, Torrey [132] argued that the role of the parietal cortex in schizophrenia has been understated, and highlighted the presumed role of the parietal cortex in auditory working memory, spatial selective attention, and especially sensory integration. In particular, the inferior parietal lobule is considered to be a recent evolutionary development and to be one of the last regions of the brain to mature, making it particularly susceptible to developmental neuropathology. In one of the first studies to demonstrate grey-matter losses in early-onset schizophrenia, Thompson et al. [133] found grey matter losses began in parietal cortex then gradually spread forward throughout the brain affecting STG and then frontal regions (See review [134]).

### Limitations of Present Study

This study employed a cross-sectional rather than a longitudinal design to study the effects of illness duration. Effects of selection bias may compromise the results. Chronic schizophrenia participants were primarily recruited from a database of research volunteers, which is likely biased towards individuals with milder symptoms and better social support than might be expected of chronic patients in general. By contrast, most of the recent-onset participants were recruited by more direct approaches within the clinical setting and provide a more representative sample of the recent-onset schizophrenia population. We did not control for possible effects of medication or the increase in cigarette consumption in the chronic patients.

We used a traditional duration-deviant oddball paradigm in which long duration deviants were randomly presented within a sequence of short standards, and which has previously been used to demonstrate reduced MMN in schizophrenia [8]. However, the exogenous response to short and long duration tones presented in isolation are different, and this confounds the measurement of MMN. Several reviews have examined this issue as well as problems associated with differential stimulus adaptation and have suggested alternative experimental paradigms that may provide better control [135], [136].

In our discussion, we should distinguish the ERP data, which are direct observations, from the *cortically-constrained* LORETA analysis, which is a *model* of the ERP data. The EEG/MEG inverse problem is widely understood to be an ill-posed mathematical problem that can only be solved by making assumptions about the nature of the underlying sources and imposing appropriate constraints. Numerous distributed source (i.e. CSD) and discrete equivalent current dipole (ECD) modelling approaches exist that differ in the constraints applied, and consequently produce different source solutions. The degree to which the constraints are physiologically plausible is the critical issue. We chose a distributed source model because prior fMRI studies have suggested multiple cortical regions were active; used MRI-derived head models for each individual; imposed cortical location and orientation constraints on the model solutions; and adjusted the LORETA smoothness constraint to ensure adequate model fit. Each of these increases the face-validity of the model obtained. However, our conclusions, particularly those concerning the role of parietal cortex and differences in onset latency across cortical regions, need to be viewed cautiously as hypotheses derived from one particular model. Replication of these results using alternative modelling procedures, and cross validation using alternative neuroimaging techniques such as fMRI or MEG is highly desirable.

### Conclusions

We examined duration MMN in recent-onset and chronic schizophrenia. Reduced MMN was observed in recent-onset patients supporting the proposal that this may be a useful index of neuropathology in prodromal schizophrenia. Cortically-constrained LORETA analysis was performed to generate a model describing cortical sources of the scalp-recorded MMN. For the early MMN response this model suggests a focal response in temporal and a weaker distributed response in parietal cortex. A frontal source emerges later as a clearly dissociable pattern. Patient data was marked by the absence of the right-hemispheric dominance seen in controls. Onset latency in secondary, but not primary, auditory cortex was delayed. This was associated with an amplitude reduction in both primary and secondary auditory cortex and also in parietal cortex. On the basis of the EEG source modelling, we propose that information processing upstream of change detection within the primary auditory cortex may be *relatively* intact, and a core deficit in schizophrenia may lie in the primary auditory cortex, or the cortico-cortico or thalamo-cortico connections leading to auditory association cortex. Comparison of the control and schizophrenia groups, as well as the correlation with GAF, also implicates the parietal cortex as a contributor to the reduced MMN seen in schizophrenia.

## Supporting Information

Table S1 **Means and standard deviations of frontal and mastoid MMN.**
(DOC)
