# Supplementary material for: Mismatch Negativity in Recent-Onset and Chronic Schizophrenia: A Current Source Density Analysis
Source: PLoS One. 2014 Jun 20;9(6):e100221. doi: 10.1371/journal.pone.0100221 (PMC4064992; doi:10.1371/journal.pone.0100221)
Supplement: Table S1 — Means and standard deviations of frontal and mastoid MMN. (DOC) [file pone.0100221.s001.doc]

Table S1. Means and standard deviations of frontal and mastoid MMN.

|  | | **Early MMN** | | | | **Late MMN** | | | |
| --- | --- | --- | --- | --- | --- | --- | --- | --- | --- |
| **Recent Onset** | |  | | | |  | | | |
|  | | F3 | F4 | M1 | M2 | F3 | F4 | M1 | M2 |
|  | *Patients* | -1.32 (1.34) | -1.24 (1.58) | 1.16 (0.97) | 1.25 (1.05) | -1.91 (1.56) | -1.89 (1.70) | 1.32 (1.29) | 1.79 (1.30) |
|  | *Controls* | -2.03 (1.12) | -2.39 (1.21) | 1.58 (0.74) | 1.59 (0.71) | -2.97 (1.87) | -3.28 (1.69) | 1.12 (1.18) | 1.61 (0.85) |
| **Chronic** | |  | | | |  | | | |
|  | | F3 | F4 | M1 | M2 | F3 | F4 | M1 | M2 |
|  | *Patients* | -1.26 (0.97) | -1.20 (0.88) | 1.01 (0.90) | 1.06 (0.63) | -1.87 (1.07) | -1.99 (1.26) | 1.26 (0.95) | 1.28 (0.70) |
|  | *Controls* | -1.74 (1.01) | -1.86 (1.20) | 1.22 (0.89) | 1.39 (1.03) | -2.43 (1.50) | -2.58 (1.58) | 0.92 (0.76) | 1.58 (1.24) |
| **Combined** | |  | | | |  | | | |
|  | | Frontal | | Mastoid | | Frontal | | Mastoid | |
|  | *Patients* | -1.25 (1.13) | | 1.11 (0.82) | | -1.92 (1.33) | | 1.40 (1.00) | |
|  | *Controls* | -1.99 (1.11) | | 1.43 (0.79) | | -2.79 (1.64) | | 1.30 (0.93) | |

Means and standard deviations of the Duration-deviant MMN recorded at frontal and mastoid electrode sites using a nose reference. Data are presented for two time windows, Early MMN (110-160 ms) and Late MMN (160-210 ms) post stimulus onset. Participants included 16 recent onset and 19 chronic schizophrenia patients and their age and gender matched controls. Also shown in the lower panel are the same data pooled across the two illness duration groups. For the pooled data, Frontal refers to the average MMN at F3 and F4, and Mastoid refers to the average MMN at M1 and M2. The pooled data were used when computing correlations with clinical measures. Note: there are no systematic differences in the standard deviation of data from control and patient groups. Also, although the mastoid response is smaller than the frontal response, the standard deviation of these is proportionately smaller. Consequently frontal and mastoid MMN data have similar signal-to-noise ratios.
